# Supplementary figures and images for: Generational Diet-Induced Obesity Remodels the Omental Adipose Proteome in Female Mice
Source: Nutrients. 2024 Sep 13;16(18):3086. doi: 10.3390/nu16183086 (PMC11435095; doi:10.3390/nu16183086)

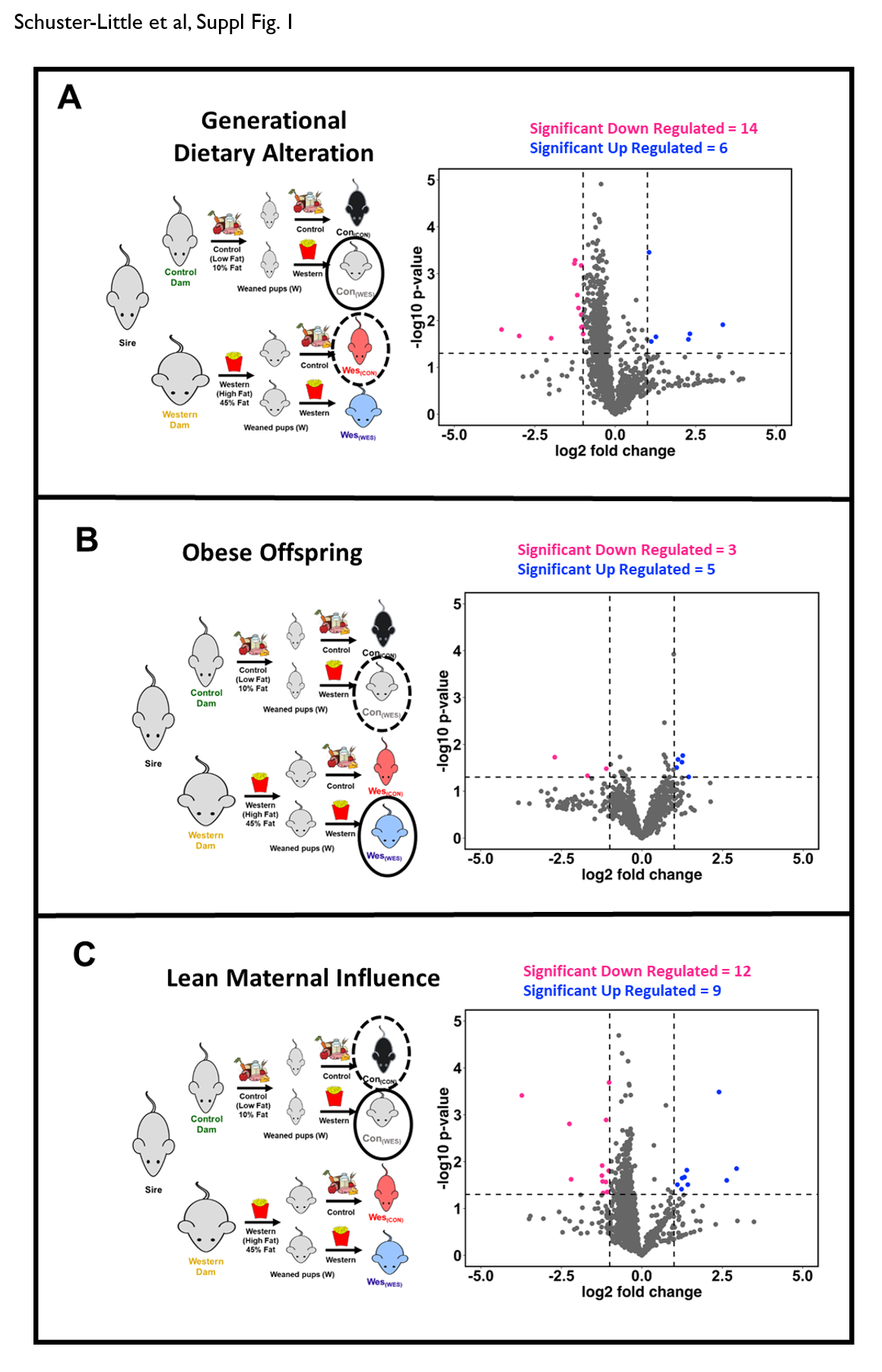

Supplement: Supplementary file 1 [file nutrients-16-03086-s001.zip › Schuster-Little et. al, Supplemental Figure S1.tif]

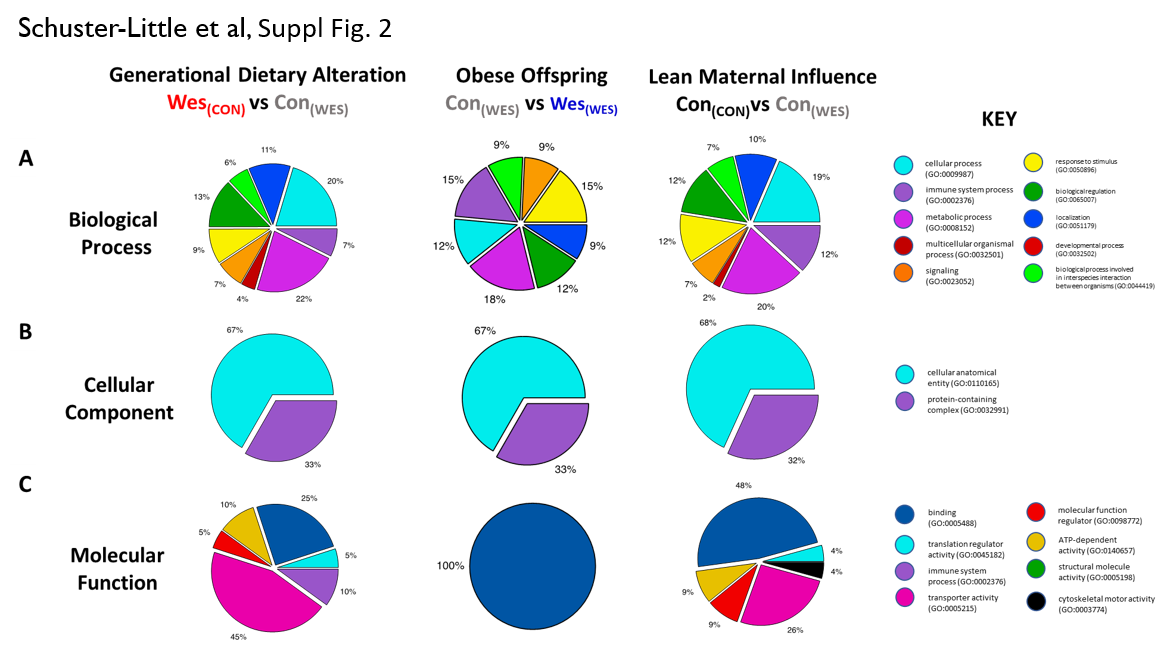

Supplement: Supplementary file 1 [file nutrients-16-03086-s001.zip › Schuster-Little et. al, Supplemental Figure S2.tif]

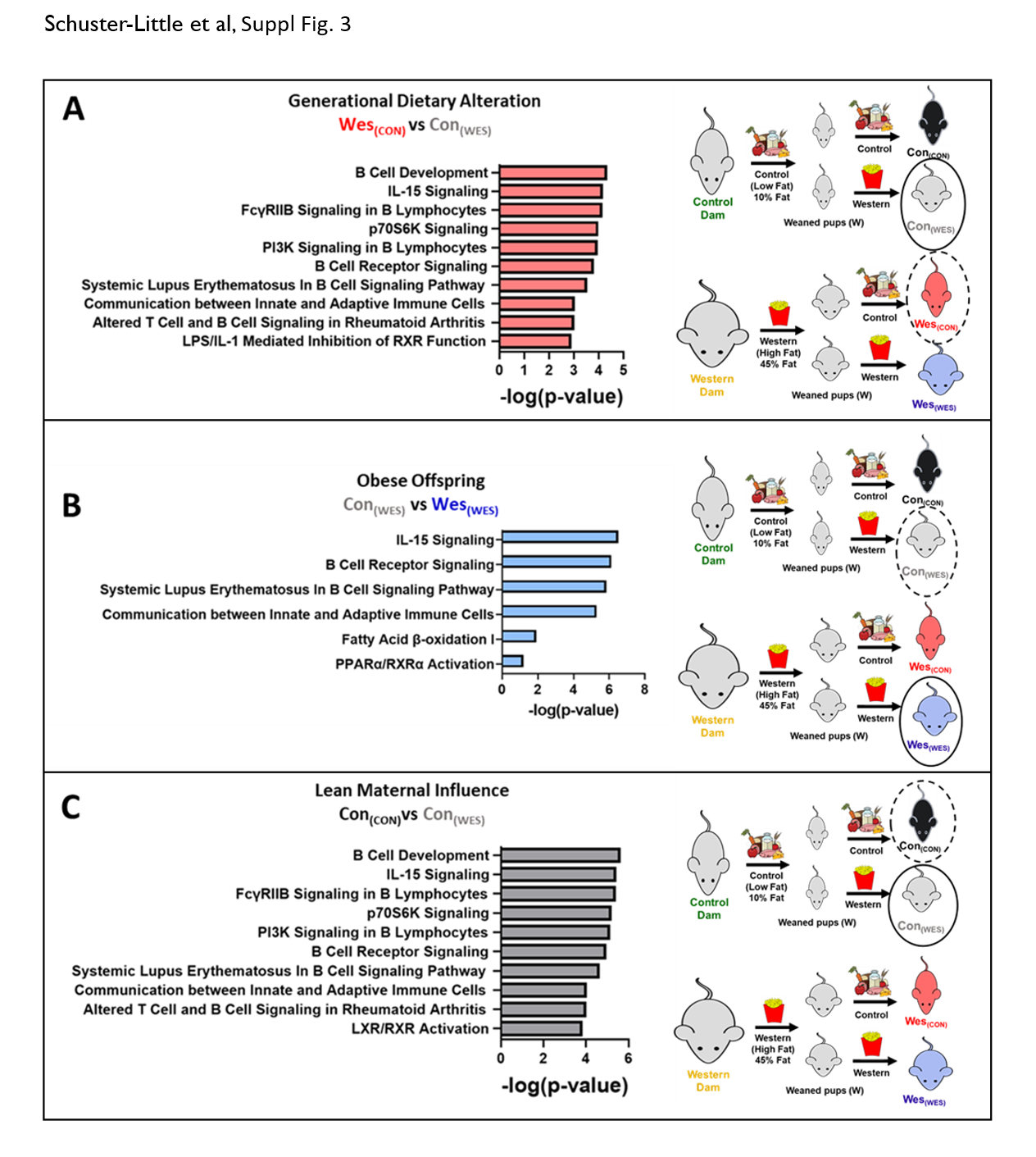

Supplement: Supplementary file 1 [file nutrients-16-03086-s001.zip › Schuster-Little et. al, Supplemental Figure S3.tif]
